# Passive Acoustic Sampling Enhances Traditional Herpetofauna Sampling Techniques in Urban Environments

**DOI:** 10.3390/s23239322

**Published:** 2023-11-22

**Authors:** Isabelle L. Barnes, John E. Quinn

**Affiliations:** Department of Biology, Furman University, Greenville, SC 29613, USA; john.quinn@furman.edu

**Keywords:** frogs, mixed methods, trail, Piedmont, unsupervised classification

## Abstract

Data are needed to assess the relationships between urbanization and biodiversity to establish conservation priorities. However, many of these relationships are difficult to fully assess using traditional research methods. To address this gap and evaluate new acoustic sensors and associated data, we conducted a multimethod analysis of biodiversity in a rapidly urbanizing county: Greenville, South Carolina, USA. We conducted audio recordings at 25 points along a development gradient. At the same locations, we used refugia tubes, visual assessments, and an online database. Analysis focused on species identification of both audio and visual data at each point along the trail to determine relationships between both herpetofauna and acoustic indices (as proxies for biodiversity) and environmental gradient of land use and land cover. Our analysis suggests the use of a multitude of different sampling methods to be conducive to the completion of a more comprehensive occupancy measure. Moving forward, this research protocol can potentially be useful in the establishment of more effective wildlife occupancy indices using acoustic sensors to move toward future conservation policies and efforts concerning urbanization, forest fragmentation, and biodiversity in natural, particularly forested, ecosystems.

## 1. Introduction

The planet and the life that inhabits it are currently undergoing a sixth mass extinction marked by extensive declines in global biodiversity. One of the taxa that has been experiencing drastic population decline is herpetofauna, namely frogs. This poses a major environmental issue as frog species are a key indicator of environmental health, and the loss of frog populations can lead to a complete food web and ecosystem collapse [1]. Thus, the need to protect and monitor frog species in the wild is becoming more and more paramount.

One of the main drivers of herpetofauna population loss is habitat loss. A clear portion of this habitat loss comes in the form of urbanization and anthropogenic expansion. Urbanization is an increasingly evident problem for biodiversity as the human population continues to grow and spread. This is becoming so much of a dilemma, in fact, that residential development is projected to increase in area by 51% between the years 2003 and 2030 [2]. This projected increase will result in a decrease in undeveloped lands in many parts of the world, leading to a decrease in biodiversity in these areas as well as a loss of the ecosystem services that nature provides [3].

Deforestation because of urbanization creates threats across taxa from vegetation to avian species, mammals, and herpetofauna. Studies by Howell et al. [4], found a significant decrease in population growth rates and stability of federally threatened Chiricahua Leopard Frogs after their natural habitat of wetlands and canyon streams was modified and removed for the implementation of development and agriculture. They found that these changes in population growth were influenced widely by habitat removal, new habitat characteristics, and the demographics that the processes of urbanization enforced upon the new patches [4]. 

Researchers have long used a diversity of techniques to sample herpetofauna [5,6]. These include a variety of active and passive sampling techniques such as transects, pitfall traps, cover boards, funnel traps, refugia tubes, and glue traps. These methods are used to reduce researcher bias and extend the length of the sampling season [7]. However, each method is limited in terms of which type of herpetofauna is being sampled (e.g., pitfall traps only work for ground-dwelling taxa). Likewise, they can be time-consuming and costly. Thus, decisions need to be made to optimize data collection [8]. In addition to these well-established methods, new sensor technologies have been developed that may provide additionality to current sampling and monitoring methods, in particular passive acoustic sampling.

Automated recording units (ARUs) are an increasingly common acoustic method to measure biodiversity in real-time and across dispersed landscapes [9]. The ability of these acoustic sensors to collect large quantities of spatial data makes them useful monitoring tools [10]. With simple programming and installation, ARUs require little time for the researcher to be in the field [10,11]. ARUs also provide researchers with the ability to cover a larger spatio-temporal scale in relation to more customary sampling practices [1]. In the last 10 years, ARUs have been widely used to monitor the occupancy, movement, and behavioral patterns of birds and bats (e.g., [12,13]) but less frequently for frogs, toads, and other herpetofauna (but see MacLaren et al. [14]).

ARUs may add additional value for detecting the distribution of herpetofauna because multiple locations can be intensively sampled concurrently. Through analysis of frog vocalizations, the relative abundance and species richness can be determined for a location, allowing an estimate of herpetofauna species diversity to be reached [1]. This can improve researchers’ ability to detect those species that may have a low detection probability or that require frequent sampling for detection [15]. In addition, the volume of data collected using ARUs opens the opportunity to leverage new machine learning tools to analyze these data, though unlike other species, for example bats, less work has been done to build effective tools for easy application when sampling herpetofauna. 

In addition to species-specific detections, the ARUs allow for a diversity of different acoustic measures of an ecosystem that can reflect the broader ecological community as compared to a single species and have been shown to correlate with other measures of biodiversity (e.g., [16,17]). These measures, frequently defined as acoustic or soundscape indices, reflect the multiple dimensions of sound in an environment, including biophony, geophony, and anthrophony. These indices can be measured as continuous variables over time, thus describing the acoustic environment a frog or other species may experience along an urban to rural gradient including other wildlife species and vehicle traffic or other sources of human noise. 

In this project, we compared traditional methods for biodiversity sampling and reporting (visual observations, citizen science, and refugia tubes) with new methods (ARUs, soundscape indices). We expected to see different, but complementary, patterns in the response of traditional and new sampling techniques, such that research on the diversity of herpetofauna in urban areas could potentially focus on a narrower set of tools and indicators.

## 2. Materials and Methods

### 2.1. Study Area

**Figure 1:**
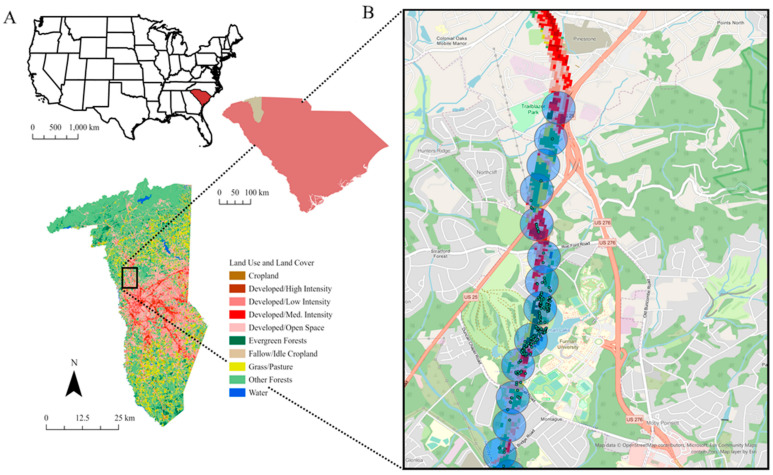
(**A**) The location of Greenville Co., SC in the southeastern United States including land use and land cover in the county as classified by the NLCD land cover types. (**B**) The same land use and land cover within 100 m of the trial and associated buffers for extracting iNaturalist data. Black circles indicate the 100 m buffer that was surveyed around the trail. The red, pink, green, and yellow pixels within the buffers indicate the land use and land cover from NLCD. The green points in the buffers indicate iNaturalist observations.

We collected data along the Swamp Rabbit Trail in Greenville County, SC, USA (Figure 1A). The county is in the Piedmont ecoregion, at the base of the Appalachian mountains, extends from northern Virginia all the way into central Alabama, and includes the northwestern corner of South Carolina, known as the Upstate. This area has experienced rapid urban development in the last century. The area has made the transition from a forest biome to a heterogenous populated forest anthrome due to the effects of human development and population growth [18]. This change has forged a novel ecological environment which mixes forest biome and urban development and has created a demand for conservation efforts to prevent further deterioration of the area. 

The Swamp Rabbit Trail (SRT, Figure 1B) is a 22-mile-long rails-to-trails development. The SRT is embedded in fragmented forested and developed areas in the South Carolina Piedmont ecoregion including urban and suburban development, subdivisions, and highway systems. The understory plant communities along the trail, not unlike many other disturbed sites in the region, are dominated by non-native and invasive vegetation. We specifically focused on six miles of the trail found adjacent to Furman University, spreading two miles northwest into the outskirts of Travelers Rest and four miles southwest towards downtown Greenville. 

### 2.2. Data Collection

We used a variety of audio and visual collection methods to determine herpetofauna presence and abundance along the trail including refugia tubes, visual observation surveys, and iNaturalist. Our particular focus for this article is the adoption of automated recording units (ARU) which are sensors that are being used with increasing frequency for a diversity of environmental monitoring efforts (as discussed above).

We placed refugia tubes every 800 m (0.5 miles) along the trail in 2018 to enhance the attainment of visual observation data. We used polyvinyl chloride (PVC) pipe traps, following the methods in Boughton et al. [19], for the collection of treefrog species. We used opaque, white PVC pipe which was cut into 60 cm lengths with a capped bottom for the refugia tubes. The tubes were mounted in trees about 1.5 m high along the trail. In the base of each tube, we poured water to create a moist environment most like those in which anurans would be found naturally [19]. To keep our study period consistent, the refugia tubes were only routinely checked during the summer months of 2021 and 2022 to align with the ARU data. 

Our observation data came from two sources. First, we conducted regular transect surveys along the trail to observe and hear individuals. Transects were 400 m along the trail and 50 m deep. Transect depth varied depending on the environment surrounding different sections of the trail. Observational surveys took place twice weekly. Second, we downloaded data from iNaturalist, filtered to Greenville, SC with only herpetofauna species selected. We included in this sample data from 2008 to the present to increase the number of observations included. As an added precaution to the quality range that can be obtained from citizen science databases, we also filtered the data to only include research-grade observations. We then used ArcGIS Pro to filter this dataset to only observations that were within 50 m of the trail.

We collected acoustic data using the SM2 from Wildlife Acoustics Inc. Recordings during the summer months (late May, June, and July) of 2021 and 2022 to ensure the optimum number of species that would be calling due to the ideal temperatures and weather [20]. Using procedures described in Sidie-Slettedahl et al. [21], we deployed the ARUs every 400 m (0.25 miles) along the trail. We hung recording devices between 1.5 and 2 m approximately 10 m off of the paved trail in a tree or shrub [22]. We programmed the ARUs at 48 dB gain (left and right) and to record for 10 min, every hour, on the hour, 24 h a day. An ARU was located at each sampling point for one week. 

### 2.3. Data Processing

To identify the frequency of detections of each species in the audio recordings we used the Kaleidoscope Version 5.3.4 software from Wildlife Acoustics (Maynard, MA, USA). Specifically, we used Kaleidoscope as a form of unsupervised classification to calculate distinct sound clusters at each recording location. We set a minimum and maximum frequency range of 150 Hz and 5500 Hz. We set a minimum and maximum length of detection at 0.1 and 15 s, 3 maximum inter-syllable gap, and 1.0 as the max distance from the cluster. These values were chosen to avoid including the repetitive call of one individual multiple times in our dataset. Details on the clustering methods can be found at wildlifeacoustics.com. We then manually inspected each file in each cluster to identify each vocalizing amphibian.

To identify spatial and temporal patterns in acoustic indices we used the tuneR package [23] for the program R (R Core Team 2019) to read sound files. We then used the soundecology [24] and seewave [25] packages to obtain values of each index from channel 1. We measured anthrophony or technophony (human-derived sounds), which we defined following the literature (e.g., [26]) as sound occurring in the 1–2 kHz range, and biophony (ecologically derived sounds), which we defined as sound occurring in the 2–8 kHz frequency range (following [26]), for each sound file. We used the soundecology and seewave packages to obtain values of Normalized Difference Soundscape Index, Acoustic Complexity Index, Acoustic Diversity Index, Acoustic Evenness Index, total entropy, and Bioacoustic Index (abbreviated NDSI, ACI, ADI, AEI, H, and BAI, respectively) for each sound file. NDSI was an index of anthropogenic noise disturbance measuring the proportion of biophony to anthrophony [26]. ACI measured the variation in the intensity of sounds [27]. ADI and AEI both measure the distribution of sound power across frequency ranges [28]. ADI quantified this distribution using the Shannon diversity index, thus measuring sound diversity similarly to species diversity, while AEI used the Gini index of evenness, thus measuring sound evenness similarly to species evenness. H was a function of temporal energy dispersal and spectral energy dispersal [25]. BAI was a function of both power and frequency range of sound between 2000 and 11,000 Hz [29]. We made three passes with this modification at 80 Hz, 1000 Hz, and 2000 Hz, following Hyland et al. [30]. Because of the filters, Biophony and BAI are considered as shown with the 2000 Hz filter as both are only measured in this acoustic space. Anthrophony and NDSI are only considered at the 1000 Hz filter, because anthrophony is measured between 1000 and 2000 Hz and thus NDSI also does not have results for 80 Hz, and the 2000 Hz filter, because it is a ratio of biophony above 2000 Hz and anthrophony between 1000 Hz and 2000 Hz.

### 2.4. Data Analysis

We used the program R 4.0.2 (R Core Team 2019) to synthesize, visualize, and analyze the data with the ggplot and dplyr packages [31]. For individual species, count per unit effort (CPU) was calculated by dividing the total number of observations by the number of data inputs recorded at each location. To avoid pseudo-replication in the soundscape data, we averaged all values for each index for each sampling. Thus, a site was the unit of study for subsequent statistical analyses of ARU data. We tested for spatial relationships using linear regression with land use and land cover as explanatory variables for both CPU and each acoustic index. Significance was based on an alpha value of 0.05.

## 3. Results

**Figure 2:**
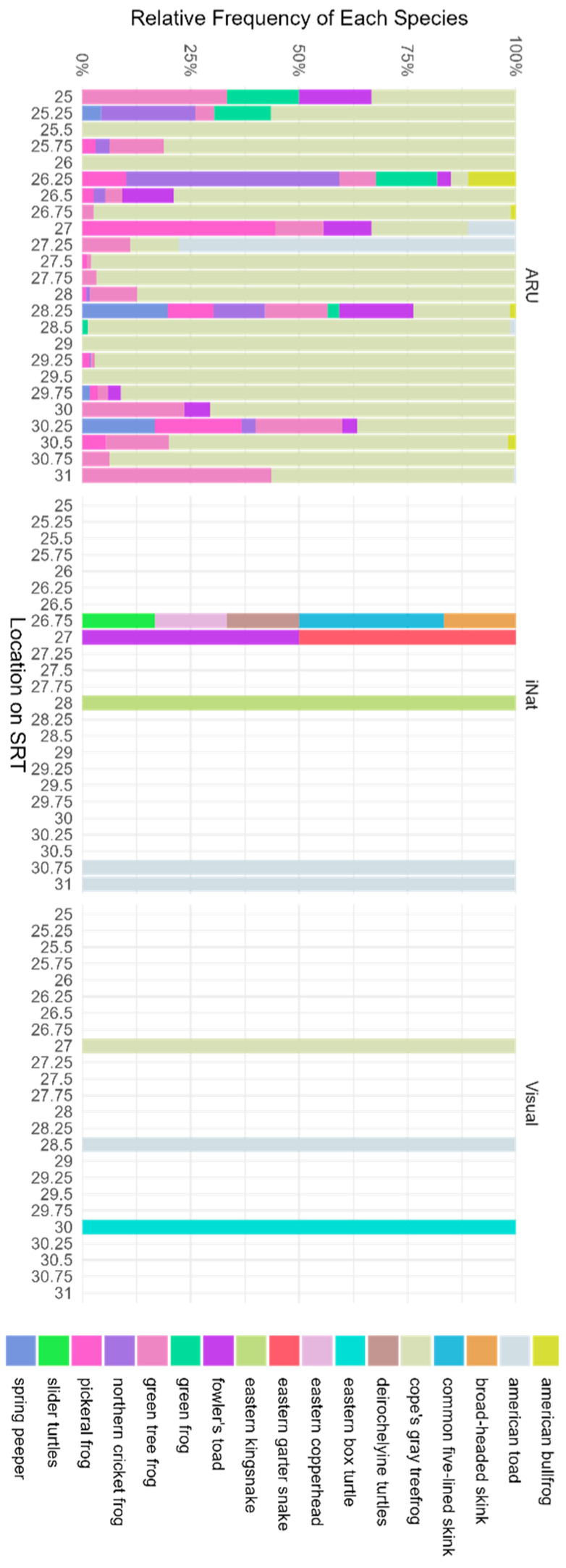
Species relative frequencies (colors) by location and observation technique. Data was collected along the Swamp Rabbit Trail, Greenville SC. ARU and visual observation data from Summer 2021 to 2022. iNaturalist data from 2016 to 2021.

In total, our different collection methods were able to detect 1419 herpetofauna observations (Figure 2; 1405 from the ARUs, 0 from refugia tubes, 11 from iNaturalist, and 3 visual observations). By including ARU data our number of detections increased by 15× and the number of species increased from 11 to 17. From the ARUs alone, we were able to accurately detect nine different anuran species: Cope’s Gray Treefrog (*Dryophytes chrysoscelis*), American Bullfrog (*Lithobates catesbeianus*), American Toad (*Anaxyrus americanus americanus*), Fowler’s Toad (*Anaxyrus fowleri*), Green Frog (*Lithobates clamitans*), Green Tree Frog (*Dryophytes cinereus*), Northern Cricket Frog (*Acris crepitans*), Pickeral Frog (*Lithobates palustris*), and Spring Peeper (*Pseudacris crucifer*). Cope’s Gray Treefrog was the most frequently detected species at most locations. The greatest number of species was detected at mile marker 28.25 on the trail with nine of our species being detected by two different data collection methods. Three species were identified by multiple collection methods. For example, the American Toad was captured with all three methods. Meanwhile, Cope’s Gray Treefrogs were found both audibly and visually through the ARU data as well as during visual transect monitoring. The Fowler’s Toads were also identified by two of the collection methods: ARUs and iNaturalist.

However, with our other detection methods, we were also able to include other, less vocal herpetofauna groups including snakes, turtles, and skinks. We totaled eight non-anuran species identifications using visual and citizen science data collection: Broad-headed Skink (*Plestiodon laticeps*), Common Five-lined Skink (*Plestiodon fasciatus*), Deirochelyine Turtles, Eastern Box Turtle (*Terrapene carolina carolina*), Eastern Copperhead Snake (*Agkistrodon contortrix*), Eastern Garter Snake (*Thamnophis sirtalis sirtalis*), Eastern Kingsnake (*Lampropeltis getula*), and Slider Turtles (*Trachemys*). Also, by restricting the iNaturalist data to only our 2-year study period, we would have lost 81.1% of our dataset from iNaturalist.

**Figure 3:**
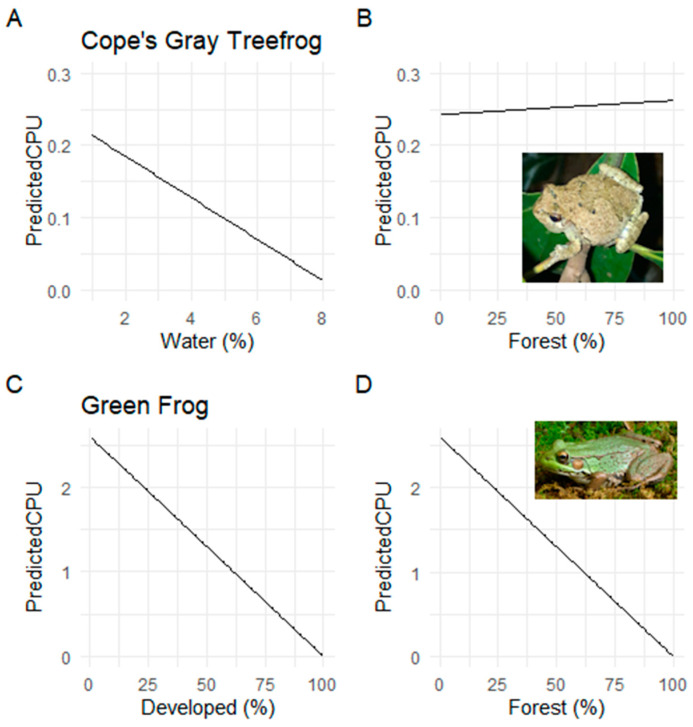
Count per unit effort (CPU) as a factor of habitat type for (**A**) Cope’s Gray Treefrog and wetland habitat, (**B**) Cope’s Gray Treefrog and forested habitat, (**C**) Green Frog and developed habitat, and (**D**) Green Frog and of forested habitat. Data collected along the Swamp Rabbit Trail, Greenville, SC, USA.

These data also allow us to observe relationships between different species and land cover types. For example, the Green Frog displayed a significant, but different, relationship with the combination of both the developed and forested cover (Table 1, Figure 3). Though not significant, the Cope’s Gray Treefrog, a species that was observed by every observation technique, showed slight associative responses between occupancy and the presence of water but lacked this response when compared to occupancy within forested environments (Table 1, Figure 3).

**Figure 4:**
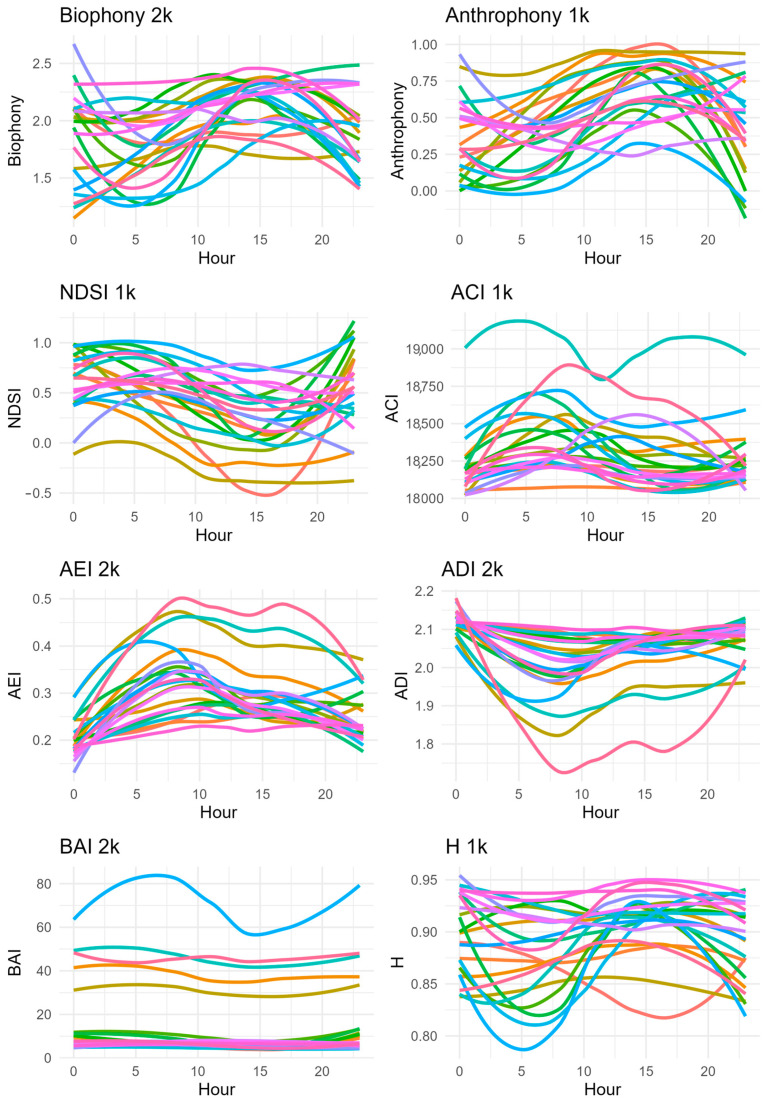
Variation in each filtered acoustic index across a 24 h time window at each location (line colors). Data collection using automated recording units (SM2 from Wildlife Acoustics) in spring and summer of 2021 and 2022. Acoustic indices calculated in R using the packages described in the methods. 1K and 2K represent the filter applied to each of the indices.

There were no statistically significant relationships (*p* > 0.20 for all indices) between the acoustic indices and measures of adjacent land use and land cover. While there were no statistical relationships between the indices and associated land use and land cover, we did find evidence of clear heterogeneity over space and time for many indices, allowing for better tracking of biodiversity over space and time. For example, both biophony and anthrophony varied between their respective minimum and maximum values between locations (Figure 4). In some locations the index value changes by nearly that same amount over a 24 h period. Perhaps of greater value are the clear outliers in the ACI and ADI and more so in the BAI that may suggest soundscapes with richer biodiversity or a greater impact of change.

## 4. Discussion

A long-standing challenge in biodiversity research, and in particular herpetological research, is the inability to conduct a comprehensive survey of a location due to the variability of species over space and time. In this case study, our results clearly show variation in the detection of individual species identified through unsupervised classification and in communities identified through acoustic indices. Focusing on the former, by leveraging sensors, ARUs clearly contributed to the greatest number of detections, thus increasing estimates of herpetofauna diversity. However, they could not pick up everything, emphasizing the necessary variations in data collection methods to conduct a comprehensive analysis of biodiversity at a point or within a region. By using different detection and identification methods, we were able to create a more comprehensive index of the study site. There were, however, vocal species that we were unable to detect via audio data due to noise congestion or other factors. An example of this can be seen in Figure 1 where the American Toad (depicted by the color gray in the figure) was not detected by the ARUs at mile markers 30.75 and 31.0 on the Swamp Rabbit Trail, but we were able to visually identify them at that location using iNaturalist citizen science data. Likewise, the variability in the soundscape indices suggests there is variation in acoustics data that could be explained by the same variables as the occupancy data or, more likely, other measures of environmental change. While we did not separate what specific species were affecting each index, the five sites that stand out for the BAI warrant further investigation for conservation efforts for herpetofauna and other wildlife. Moreover, if there is evidence of a correlation between the BAI (or another index) and richness or occupancy of key herpetofauna, overall or at a given point in time, it may be that these indices can be used to track herpetofauna diversity until better machine learning tools become available to process these data.

As stated, each study method contains strengths and weaknesses. Some of these weaknesses were highlighted in data processing and analysis. Active sampling, through transect sampling and physical searching for taxa, allows for an increased probability of sample bias due to the likelihood of the researcher only looking in locations in which herpetofauna species are more likely to be found. Alternatively, passive sampling made possible with sensors embedded in ARUs reduce bias but also restrict the comprehensiveness of the study due to the specifications for a distinct type of taxa. This was seen through the lack of non-vocalizing species that were detected using ARUs, an expected result. Lastly, citizen science data has the potential to be an unreliable research source due to the likelihood of the public misidentifying an organism; however, it allows researchers to gain a larger understanding of species found both spatially and over a longer time scale. This potential for error and a less reliable dataset can be counteracted using filters, such as scientific-grade observations, within citizen science applications; however, this also greatly reduces the available dataset that the platform has to offer. Citizen science data is also restrictive as it may not include rare or elusive species in its observational skillset if a species had not been recorded prior.

Like ARUs, citizen science data collection has increased in frequency and usefulness. For ecology, this growth has been driven by the popular citizen science platform iNaturalist, which was launched in 2008 (inaturalist.org). Platforms such as iNaturalist allow researchers to surpass the confines of time, effort, and funding due to the immense volume of field observations that can be gathered by the larger public [32]. Through using data from iNaturalist, we were also able to include species that may have evaded our efforts in data collection despite their presence in the study site in our comprehensive data set. Many of these included species do not typically rely on sound and calls for communication, like skinks, salamanders, snakes, and turtles. Even though some of these groups may use acoustic communication, like a snake hissing or rattling, these vocalizations were not detected by our ARUs due to the greater amplitude of the surrounding forest noise. By using iNaturalist data, we were also able to account for species that may have been observed outside of our study timespan. Instead of only including data from the summer months of 2021 and 2022, we also were able to include observations from as far back as August 2016 to increase the comprehensiveness of our study.

One challenge with the ARU data that persists is the necessity of manual identification of species. We attempted to build an advanced classifier within Kaleidoscope. However, due to the lack of regularity of the individual species’ vocalizations (a short chirp versus a call) and the inevitable variability of background noise overpowering the vocalizations themselves, we were unsuccessful. Future work could be oriented toward the formation of an efficient, advanced classifier for herpetofauna species similar to the ones that are currently seen for birds and bats. We also experienced shortcomings with our refugia tube collection method. Due to the lack of observations from these refugia tubes, we can only assume that the frogs had a bias to continue to choose natural refugia over an artificial one [33]. Thus, we concluded that refugia tubes were not a useful data collection method for our study site.

## 5. Conclusions

In conclusion, in this case study, the use of a variety of different collection methods led to a more comprehensive study of the herpetofauna along the study transect. This allows us to think further about how these relationships may vary seasonally and develop further occupancy patterns. Locally, this information can be used to further implement conservation strategies regarding the establishment of an effective buffer around the trail and the forested areas through which it runs. Future research should evaluate the added value of using multiple techniques, including ARUs, in other study regions. In addition, researchers should continue to use this variety of collection methods to create a reliable index for other species.

## Figures and Tables

**Table 1:** Regression estimates and standard error for the response of Cope’s Gray Treefrog, Green Frog, and total species abundance, as a function of the percentage of water, forest, and development along the Swamp Rabbit Trail. Significant *p* values noted in bold and shown in Figure 3.

	Cope’s Gray Treefrog			Green Frog			Total		
	Estimate	Std. Error	*p* Value	Estimate	Std. Error	*p* Value	Estimate	Std. Error	*p* Value
Intercept	0.243	0.120		2.599	0.139		0.054	0.035	
Water (%)	−0.029	0.024	0.250	−0.001	0.001	0.800	0.000	0.001	0.800
Forest (%)	0.000	0.002	0.920	**−0.026**	**0.001**	**0.0003**	−0.001	0.001	0.320
Developed (%)	0.001	0.003	0.810	**−0.026**	**0.001**	**0.0003**	0.001	0.001	0.255

## Data Availability

Data will be accessible within Harvard Dataverse following publication of the research and accompanying manuscripts.
